# Emergence of *Rhodotorula mucilaginosa* among pet animals: a possible public health risk on the move

**DOI:** 10.1186/s12866-025-03894-9

**Published:** 2025-05-07

**Authors:** Hassan Aboul-Ella, Tarek Mosallam, Ojena Samir, Aisha Ali, Arwa Qasim, Hossam Eldin Mahmoud, Ahmed Samir

**Affiliations:** 1 Department of Microbiology, Faculty of Veterinary Medicine, Cairo University, Giza, Egypt; 2 Animal Reproduction Research Institute, Haram, Giza, Egypt; 3 LeptoVet Laboratory for Veterinary Diagnostic Services, Garden City, Cairo Egypt

**Keywords:** Cross-kingdoms talk, Otomycosis, Nasal mycosis, Co-infection, Polymicrobial infection, Emerging fungal infection

## Abstract

Rhodotorulae are environmentally ubiquitous yeasts that were originally considered non-pathogenic. However, over the last fifty years, *Rhodotorula mucilaginosa* (*R. mucilaginosa*) has established itself as an emerging opportunistic pathogen incriminated in several systemic and localized infections in humans and animals. Most of the rhodotorulosis cases were linked directly or indirectly to an immunocompromising event in the affected cases. Nevertheless, recently, a few non-immunocompromised cases were reported. In the current study, performed throughout the period from April/2023 to April/2024, 450 samples were collected from pet animals and investigated for the existence and coexistence of *Rhodotorula* spp. in different clinically diagnosed infectious cases. 173 (38.5%) samples showed positive direct microscopic slides of different sizes of Gram-positive budding yeast cells, 21 (4.7%). *Rhodotorula* isolates mixed with other yeasts and/or bacterial pathogens were recovered from nasal passages and ear canal swabs collected from dogs and cats suffering from nasal affection and otitis externa. Laboratory investigations were based on sample collection, microscopic examination, primary isolation and identification, biochemical and post-culturing characterization, antifungal susceptibility testing, VITEK 2 Compact Identification System, DNA extraction, PCR amplification, sequencing, and phylogenetic analysis. Moreover, antifungal susceptibility testing based on the standard broth microdilution test was applied to the recovered *Rhodotorula* isolates. In conclusion, the present findings spotlighted a prospective insight into the role of the emergence of *R. mucilaginosa* among pet animals and its possible public health concerns.

**Clinical trial number**: Not Applicable.

**Supplementary Information:**

The online version contains supplementary material available at 10.1186/s12866-025-03894-9.

## Background

Hippocrates was the first to describe a yeast infection caused by *Candida albicans* in the fifth century and named it “thrush” [1]. Since then, the primary and the most frequently isolated yeast associated with human and animal infections is *Candida albicans*. Non-albicans *Candida* and other new and emerging yeast agents have been demonstrated in various forms and types of disease, some of which are systemic life-threatening and others are local superficial conditions [2, 3]. Despite the recognition of different yeasts as agents of disease, little medical or scientific concern was given to them, in contrast to the many serious and highly prevalent bacterial infections recognized in the late 1800s. However, the advent in the 1960s of new modalities to treat cancer, increasing use of central venous catheters, an explosion in new antibacterial agents’ development, increases in average life expectancy, and other medical developments soon paved the way for innocuous yeasts to cause serious infections.

With further developments in medical intervention, increasing the population of immunosuppressed and immune-deficient patients, and raising animal-human interaction habitual occasions, the list of yeasts that can cause disease continues to grow. Disturbances of the microbiome niche environment and conditions that adversely affect the host immune system predispose and facilitate the pathogenicity transition of opportunistic microbes and emerging new infectious diseases [4]. Factors predisposing the human or animal host to fungal infection establishment are long-term or repeated prescription of broad-spectrum antibiotics, impairments of epithelial barriers especially that reflect on the skin and the gastrointestinal tract e.g., by chemotherapy, surgical interventions, or catheter-based medical processes, and treatment with immunosuppressive drugs such as corticosteroids [5, 6]. *Rhodotorula* spp. are saprophytic yeasts that are usually isolated from dairy products and dumpy or moist environmental sources including bathroom surfaces, swimming pools, and planted places as commensal inhabitants of the gastrointestinal tract and skin of mammals [7–9].

Reported infections with *Rhodotorula* spp. in pet animals are rare and usually confirmed by laboratory isolation, identification, antifungal susceptibility testing, and the degree of treatment response during the case follow-up [9, 10]. Opportunistic *Rhodotorula* infection of humans, which is known as rhodotorulosis, is increasing in predisposed immunocompromised cases and is associated with high mortality rates despite interceptive antifungal treatments [11–13]. Therefore, rhodotorulosis showed an increasing concern as a zoonotic threat that can be transmitted directly to susceptible humans from infected or apparently healthy companion animals, or to a patient from a medical occupation member who raises an infected pet animal [14], or indirectly through medical equipment manipulation such as catheters and other medical prosthetic transplants [15].

Nowadays, fungal infections, whether zoonotic or sapronotic, are increasingly important to public health worldwide [16]. A number of these infections are due to established potentially pathogenic fungal agents such as dermatophytosis [17–20], histoplasmosis [21], and candidiasis [5, 16]. On the other hand, it is notable that some emerging opportunistic pathogens with zoonotic potential have inadequate attention by international public health efforts, leading to insufficient attention to their precautionary measures [22]. Therefore, the current study highlighted the incrimination of one of those neglected causes; *R. mucilaginosa* in several mixed infection cases among pet animals.

## Materials and methods

### Study design

From the practical experience of our research team as veterinary clinical mycologists, an uprising in the laboratory isolation of carotenoid-pigmented yeast colonies has been noticed since 2021 and upwards. All obtained isolates during this observational period were in mixed infection circumstances such as yeast/yeast, yeast/bacteria, and yeast/yeast/bacteria polymicrobial isolates. The current study was designed to investigate the clinical incrimination of *Rhodotorula* spp. among pet animals with a prospective insight into the cross-kingdom talk infection models through the following steps: sample collection, microscopic examination, primary isolation and identification, biochemical and post-culturing characterization, antifungal susceptibility testing, VITEK 2 Compact Identification System, DNA extraction, PCR amplification, sequencing, and phylogenetic analysis.

### Samples collection

A total of 450 different clinical samples (ear swabs, vaginal swabs, nasal swabs, skin swabs, urine samples, fecal samples, and semen samples) were collected from the two widespread companion animal species (dogs and cats) of different ages and diverse immune status. All samples were collected from active infection cases that are presumptively and differentially clinically diagnosed as active infection cases by an expert veterinarian. All samples were obtained from April/2023 to April/2024 in licensed veterinary clinics, in the Greater Cairo Area (GCA), Egypt. As an integral part of these veterinary clinics’ diagnostic protocols, informed consents were obtained from the owner of the animals. All samples were collected by standardized sample protocol applied by expert Egyptian veterinarians and submitted to LeptoVet Laboratory for Veterinary Diagnostic Services (an ISO9001 accredited and licensed veterinary laboratory), Egypt for further laboratory bacteriological investigations (the bacterial isolation and the antimicrobial sensitivity tests results were reported after 3 days from the sample receiving date and based on it the responsible clinician prescribed the tentative treatment protocol) and to the Mycology Laboratory, at the Department of Microbiology, Faculty of Veterinary Medicine, Cairo University, Egypt for further laboratory mycological investigations (the fungal isolation and the antifungal sensitivity tests results were reported after 14 days from the sample receiving date and upon which the curative treatment protocol was prescribed for those non-responders to the tentative protocol).

Other samples were collected 3–4 weeks post antifungal application from those cases where *Rhodotorula* spp. was primarily isolated to ensure the elimination of the fungal agent.

### Microscopic examination

The microscopic examination has been performed in two different stages throughout the current work:

- Pre-culturing: all samples were subjected to direct microscopic examination by standard Gram’s technique by light microscope (oil immersion lens) as a presumptive indication of a yeast infection with the determination of the cell shape and type of budding.
- Post-culturing: all carotenoid-pigmented colonies on corn meal tween 80 agar plates were further microscopically examined by light microscope (low and high dry powers lenses). Spheroidal to oval budding cells without the rudimentary formation of hyphae were a sign to exclude *Sporobolomyces* spp. involvement [23, 24].

### Primary isolation and identification

Culture-based isolation and identification have been performed in two different stages throughout the current work:

- Throughout the primary isolation protocol (directly following receiving the samples at the mycology laboratory): Whatever the result of the pre-culturing direct microscopic examination was, all samples were subjected to the subsequent isolation step. The samples were quadrantally streaked on chloramphenicol-supplemented Sabouraud’s dextrose agar (SDA, HIMEDIA, India), incubated at 25 °C for 3–5 days [25], then kept incubated with daily observation for 3 weeks. The carotenoid-pigmented colonies obtained were subjected to post-culturing microscopic examination followed by the subsequent identification and characterization techniques; biochemical and post-culturing characterization, antifungal susceptibility testing, VITEK 2 Compact Identification System, DNA extraction, PCR amplification, sequencing, and phylogenetic analysis.
- Throughout the secondary follow-up protocol (3–4 weeks post-antifungal treatment applications):

The samples were quadrantally streaked on chloramphenicol-supplemented SDA (HIMEDIA, India) and incubated at 25 °C for 3–5 days [25].

### Biochemical and post-culturing characterization

All the following testing procedures were performed on freshly sub-cultured colonies and were repeated in duplicates to ensure the reproducibility of the obtained results [26–28]. Catalase test [29], oxidase test [30], bile esculin test [29], urease test [31], nitrate reduction test [32], sugar assimilation tests (Modified rapid tube carbohydrates assimilation [33] and Auxanographic carbohydrates assimilation [34]), modified nitrate assimilation test [35], temperature survivability tests [36], cycloheximide (CHX) resistance test [37], blood agar plate (BAP) test [38], lipolytic activity on Tween 80 agar [39], micromorphology (Pseudo-hyphae and chlamydospores) demonstration on corn-meal Tween 80, rice-meal Tween 80 agar, and potato-meal Tween 80 agar [40], capsule demonstration using India ink [41], and broth culture growth pattern and pellicle formation test [42].

### Antifungal susceptibility testing

Antifungal susceptibility testing was performed using the broth microdilution method and the outcome results were evaluated and interpreted according to the latest clinical and laboratory standard institute (CLSI) manual (M27M44S 3rd ed, 2022) [43]. Six antifungals belonging to four different classes (Polyenes; Amphotericin B, Echinocandin; Micafungin, Caspofungin, Azoles: Fluconazole, Voriconazole, and Pyrimidine; Flucytosine) were used. The selection of the antifungal panel to be tested through the performed antifungal susceptibility tests depended on certain criteria; the updated CLSI guidelines, scholarly internationally published articles in the same field of the current study, commercial availability of the used active principle, and the availability of a proper active principle form to be prescribed to pet animals. The antifungal agents and concentrations tested were as follows: 0.06 to 16 µg/mL for Amphotericin B (AmB, SIGMA-ALDRICH, USA), 0.12 to 128 µg/mL for Fluconazole (FCZ, SIGMA-ALDRICH, USA), Voriconazole (VOR, SIGMA-ALDRICH, USA), 0.06 to 32 µg/mL for Caspofungin (CAS, SIGMA-ALDRICH, USA), 0.06 to 32 µg/mL for Micafungin (MICA, SIGMA-ALDRICH, USA), and 0.06–64 µg/mL for Flucytosine (5-FC, SIGMA-ALDRICH, USA).

A few colonies from each tested isolate were picked and suspended in 5mL sterile saline. The resulting suspension was vortexed and its density was adjusted to 0.5 McFarland standard. A working suspension is made by adding 20µL of stock yeast suspension to 11mL of RPMI 1640 with 1.5% dextrose, pH 7.0. 100µL of the working suspension is distributed in all antifungal-carrying tested wells in the microtiter plate. The microplates were incubated at 35 °C for 48 h. The MICs were determined visually after 24 h and 48 h of incubation. MIC endpoint for Amphotericin B was the lowest tested drug concentration able to prevent any visible growth. The MICs for the rest used antifungals were based on a prominent decrease (≥ 50%) in growth.

### VITEK 2 compact identification system

The obtained carotenoid pigment-yeast isolates were also re-identified using the VITEK 2 Compact Identification System with the specialized yeast identification card (VITEK 2 Yeast ID; BIOMERIEUX, Marcy l’Etoile, France) following the manufacturer’s instructions. The input inoculums were taken from 48-h subcultures on chloramphenicol-supplemented SDA plates. All inocula were compared and adjusted to the McFarland standard no. 2 using an ATB 1550 densitometer (BIOMERIEUX, Marcy l’Etoile, France) by suspending a yeast colony in 0.45% aqueous sodium chloride (NaCl) solution (BIOMERIEUX, Marcy l’Etoile, France). The VITEK ID-YST card consists of 64 wells with 46 fluorescent biochemical tests measuring carbon source utilization, nitrogen source utilization, and enzymatic activities. The four subsidiary tests are actidione, phosphatase, urea, and nitrate. The VITEK 2 system recommends straightforward additional tests in identification situations with low discrimination (most of them can be completed on the same day). Additional tests were chosen to show sporangia, polysaccharide capsules, convoluted colonies, hyphae or pseudohyphae, a carotenoid pigment, blastospores, growth at 37 °C, and growth without oil. The cards were automatically filled, sealed, and transported into an incubator at a temperature of 35 °C using the integrated VITEK 2 machine. The cards were automatically submitted to fluorescence measurement every 15 min. A particular algorithm was used to interpret each profile. The 18-hour incubation period was followed by comparing the profile result with the ID-YST database [44].

### DNA extraction

Three representative isolates, one representing each phenotypic cluster, were selected for DNA extraction, PCR amplification, sequencing, and phylogenetic analysis.

To extract DNA from the three representative isolates, a colony was picked from the SDA agar, suspended in sterile water, thoroughly mixed, and centrifuged at 14,000 rpm for 10 min. Then, the supernatant was discarded, 300µL of lysis buffer was added to the biomass and incubated at 500 rpm at 37℃ for 1 h. After incubation, 200µL of Tris EDTA (TE) buffer was added and each sample was mixed for 1 min. 200µL of phenol: chloroform: isoamyl alcohol (25:24:1), pH 8.0 was added and mixed for 1 min. The samples were centrifuged for 10 min at 14,000 rpm. The top layer was collected and 600µL of cold 96% ethanol was added and mixed for 30 s. The prepared samples were incubated at 18 °C for 30 min and centrifuged at 14,000 rpm for 10 min. The supernatant was discarded, 500µL of 70% ethanol was added, and the mixture was centrifuged under the same conditions. Finally, ethanol was removed and the extracted DNA was resuspended in 25µL of sterile nuclease-free water [45]. The extracted DNA’s quantity, purity, and intactness were evaluated by NanoDrop spectrophotometer (THERMOFISHER-SCIENTIFIC, USA) and gel electrophoresis (CLEAVER-SCIENTIFIC, UK), respectively.

### PCR amplification, sequencing, and phylogenetic analysis

The PCR reaction for sequencing was performed using universal primers, ITS4: Reverse (R), (5’-TCCTCCGCTTATTGATATGC-3’) and ITS5: Forward (F), (5’-GGAAGTAAAAGTCGTAACAAGG-3’), for ITS region including 5.8 S rRNA region [46]. Amplification condition was performed in a 20µL reaction volume and consisted of 2µL 10x Taq PCR buffer, 1.6µL of 2.5 mM dNTPs mixture, 1µL of 10pmole/µL of each primer (F and R), 1.5µL template (20 ng/µL), and 0.2µL KOMA-Taq (2.5 U/µL), then complete up to the final reaction volume (20µL) using distilled water (HPLC grade). The thermal cycler was programmed as follows; 1 cycle of 5 min at 95 °C for initial denaturation, followed by 30 cycles of (30 s at 95 °C for denaturation, 2 min at 55 °C for annealing, 90 s at 68 °C for extension), and finally 1 cycle of 10 min at 68 °C for the final extension. Unincorporated PCR primers and dNTPs were removed using a Montage PCR clean-up kit (MERCK MILLIPORE, USA). The purified PCR products were then sequenced using the ITS5 and ITS4 primers. Sequencing was performed using Big Dye terminator cycle sequencing kit v.3.1 (APPLIED BIOSYSTEMS, USA). Sequencing products were resolved on an Applied Biosystems model 3730XL automated DNA sequencing system (APPLIED BIOSYSTEMS, USA) at (MACROGEN, Inc., Seoul, Korea).

## Results

### Microscopic examination

- Pre-culturing: 173 samples showed positive direct microscopic slides of different sizes of Gram-positive budding yeast cells.
- Post-culturing: 21 carotenoid-pigmented isolates showed spheroidal to oval budding cells without the rudimentary formation of hyphae.

### Primary isolation and identification

- Throughout the primary isolation protocol (directly after receiving the samples at the mycology laboratory): 21 carotenoid-pigmented isolates were obtained and presumptively identified as *Rhodotorula* spp. Phenotypically those isolates have been categorized into three clusters; cluster 1: heavily and rapidly growing coral pink smooth moist colonies (9 isolates), cluster 2: scarcely and slowly growing salmon pink mucoid colonies (7 isolates), and optimally growing orange dry colonies (5 isolates). The case and clinical history for those cases are collectively described and presented in Tables 1 and 2.
- Throughout the secondary follow-up protocol (3–4 weeks post-antifungal treatment applications): All retested samples showed no growth of any fungal agents.

**Table 1:** A collective representation of the collected data about each involved case including the obtained samples and the obtained isolates

	Age (years)	Species	Gender	Receiving date	Infection site	Mixed infection	Immunocompromising associating event over the last 6 months	The preculturing microscopic examination results regarding the presence of Gram-positive budding yeast cells	Phenotypic cluster
**S1**	8	Dog	F	4/April/2023	Ear canal	*Candida albicans* and *E. coli*	prolonged antibiotic administration, improper poor nutritional regimen, and outdoor housing	+	1
**S2**	6	Dog	F	20/April/2023	Ear canal	*Proteus mirabilis*	prolonged antibiotics administration, recurrent chronic cystitis, outdoor playing allowance, and pregnancy	+	1
**S3**	13	Dog	M	6/May/2023	Ear canal	*Staphylococcus aureus*	prolonged antibiotic administration, and outdoor garden-walking allowance	+	2
**S4**	7	Dog	F	10/May/2023	Ear canal	*Staphylococcus aureus*	prolonged antibiotic administration	+	1
**S5**	9	Cat	F	25/June/2023	Nasal passage	*Pseudomonas aeruginosa*	prolonged antibiotic administration	+	1
**S6**	12	Dog	F	7/July/2023	Nasal passage	*Staphylococcus aureus*	prolonged antibiotic and corticosteroid administration	+	2
**S7**	11	Dog	M	22/July/2023	Ear canal	*Pseudomonas aeruginosa*	prolonged antibiotic administration, outdoor housing	+	3
**S8**	6	Cat	M	23/July/2023	Ear canal	*Staphylococcus aureus*	prolonged antibiotic administration	+	1
**S9**	**5.5**	**Dog**	**F**	**25/July/2023**	**Ear canal**	***Pseudomonas aeruginosa***	**prolonged antibiotic administration**,** outdoor housing**,** pregnancy**	**+**	**1**
**S10**	10	Cat	F	1/August/2023	Ear canal	*Pseudomonas aeruginosa*	prolonged antibiotic administration	-	1
**S11**	9	Dog	M	3/August/2023	Ear canal	*Candida albicans* and *Pseudomonas aeruginosa*	prolonged antibiotic and corticosteroid administration	+	3
**S12**	6.5	Dog	F	17/August/2023	Nasal passage	*E. coli*	prolonged antibiotic administration, outdoor housing	+	1
**S13**	9	Dog	M	21/August/2023	Ear canal	*Candida albicans and proteus mirabilis*	prolonged antibiotic and corticosteroid administration	+	3
**S14**	**8**	**Dog**	**F**	**15/September/2023**	**Ear canal**	***Staphylococcus aureus***	**prolonged antibiotic administration** **and poor nutritional regimen**	**+**	**2**
**S15**	11	Cat	F	11/October/2023	Ear canal	*Pseudomonas aeruginosa*	prolonged antibiotic administration	+	1
**S16**	8	Dog	F	18/October/2023	Ear canal	*Pseudomonas aeruginosa*	prolonged antibiotic and corticosteroid administration	+	2
**S17**	8	Dog	M	1/November/2023	Ear canal	*Staphylococcus aureus*	prolonged antibiotic administration and obesity	-	2
**S18**	10	Dog	F	12/December/2023	Ear canal	*Pseudomonas aeruginosa*	prolonged antibiotic and corticosteroid administration	+	2
**S19**	**12**	**Cat**	**F**	**27/January/2024**	**Nasal passage**	***Staphylococcus aureus***	**prolonged antibiotic administration**	**+**	**3**
**S20**	7	Dog	F	19/March/2024	Ear canal	*E. coli*	prolonged antibiotic administration and outdoor activities allowance	+	3
**S21**	8	Dog	M	27/April/2024	Ear canal	*Pseudomonas aeruginosa*	prolonged antibiotic administration	+	2

**Table 2:** A collective representation, in percentages, of the case and clinical history of each case involved in the current study

Characteristic			Value (%)
Age	Puppy/kitten (up to 1 year)		0
	Adult (up to 6 years)		14.3
	Senior (up to 12 years)		85.7
	Geriatric (> 13 years)		No geriatric cases have been received throughout the study period
Host	Dog		81
	Cat		19
Sex	Male		33
	Female		77
Underlying immune affecting managemental, medicinal, physiological, or pathological condition(s)	Present		100
	Absent		0
Previous drug administration	Antibiotics		100
	Corticosteroids		23
The co-infecting bacterial and/or fungal pathogen	Bacteria	*Pseudomonas aeruginosa*	42.9
		*Staphylococcus aureus*	33.3
		*Escherichia coli*	14.3
		*Proteus mirabilis*	9.5
	Fungi	*Candida albicans*	14.2

### Biochemical and post-culturing characterization

The biochemical testing performed gave the same results for all tested isolates. Based on these results, the obtained isolates can be identified as *R. mucilaginosa*. The result of each performed biochemical and further post-culturing characterization technique is summarized in Table 3.

**Table 3:** Summarization and tabulation of the result of the different biochemical tests performed on all obtained isolates

Biochemical test			Result
Catalase			+
Oxidase			-
Bile esculin			-
Urease			+
Nitrate reduction			-
Sugar Assimilation tests	Modified rapid tube carbohydrates assimilation		+
	Auxanographic carbohydrate assimilation		
Modified nitrate assimilation test			+
Temperature survivability test		5 °C	+
		25 °C	+
		30 °C	+
		35 °C	+
		40 °C	+
		45 °C	+
		50 °C	+
		55 °C	+
		60 °C	-
Cycloheximide resistance test			+
Blood agar plate test			-
Lipolytic activity on Tween 80 agar			-
Micromorphology			Blastospores
Capsule demonstration test			+
Broth culture growth pattern and pellicle formation test			Sediment after 48 h incubation and remain as pellicle after 10 days of refrigeration

### Antifungal susceptibility testing

**Fig. 1:**
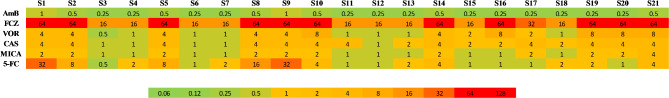
Heatmap representing the antifungal susceptibility profile of each obtained isolate. AmB; Amphotericin B, FCZ; Fluconazole, VOR; Voriconazole, CAS; Caspofungin, MICA; Micafungin, and 5-FC; Fluocytosine. (S1-S21) representing the 21 obtained *R. mucilaginosa* isolates

The results showed diverse levels of antifungal resistance: 100% were resistant to Fluconazole, Caspofungin, and Micafungin; 95% were resistant to Voriconazole; and 9.5% were resistant to Flucytosine. The antifungal susceptibility testing results for each obtained isolate against the six tested antifungals are clearly described separately in Fig. 1 and cumulatively in Table 4.

**Table 4:** Cumulative percentages (%) of the obtained isolates in correlation to the different concentrations of the tested antifungals (µg /mL)

	0.06–0.12	0.25	0.5	1	2	4	8	16	32	64	128
**AmB**	-	62	28.5	9.5	-	-	-	-	-	-	-
**FCZ**	-	-	-	-	-	-	-	42.8	4.8	52.4	-
**VOR**	-	-	4.8	33.3	9.5	28.5	23.8	-	-	-	-
**CAS**	-	-	-	23.8	19	57.2	-	-	-	-	-
**MICA**	-	-	-	47.6	47.6	4.8	-	-	-	-	-
**5-FC**	-	-	4.8	33.3	23.8	14.3	9.5	4.8	9.5	-	-

AmB; Amphotericin B, FCZ; Fluconazole, VOR; Voriconazole, CAS; Caspofungin, MICA; Micafungin, and 5-FC; Flucytosine

### VITEK 2 compact identification system

The result obtained by the VITEK 2 Compact Identification System was identical for all tested isolates. The system identified all isolates as *R. mucilaginosa*. A detailed presentation of each test result within the system is collected and tabulated in Table 5.

**Table 5:** Summarization and tabulation of the VITEK 2 compact identification system test results for all isolates obtained

Test	Result	Test	Result
LysA	**+/-**	NAGA1	-
TyrA	+	XLTa	+
dGLUa	+	dTREa	+
dRAFa	+	dXYLa	+
IRHAa	+	2KGa	-
dTURA	+	LeuA	+
IGLTa	+	ARBa	+
IPROa	+	MAdGa	+
IMLTa	-	dMNEa	+
BNAG	-	dSORa	+
LACa	+	NO3a	-
ESC	-	GRTas	-
LATa	-	dGALa	+
NAGa	-	GGT	**+/-**
ARG	+	dMLZa	+
AMYa	+	URE	+
dCELa	+	dGATa	-
dMELa	+	CITa	-
SACa	+	GLYLa	+
lARAa	+	GENa	+
ACEa	+	dMALa	+
dGNTa	-	ISPEa	+
ERYa	-	AGLU	+

They include 36 assimilation tests that are divided into carbohydrate assimilations, organic acid assimilations; L-malate (lMLTa), erythritol (Erya), glycerol (GLYa), arbutin (ARBa), amygdalin (AMYa), D-galactose (dGALa), gentobiose (GENa), D-glucose (dGLUa), lactose (LACa), methyl-A-D-glucopyranoside (MAdGa), D-cellobiose (dCELa), D-maltose (dMALa), D-raffinose (dRAFa), D-mannose (dMNEa), D-melibiose (dMELa), D-melezitose (dMLZa), L, sorbose (lSBEa), L-rhamnose (lRHAa), xylitol (XLTa), D-sorbitol (dSORa), saccharose/sucrose (SACa), D-turanose (dTURa), D-trehalose (dTREa), L-arabinose (lARAa), D-galacturonate (dGATa), L-glutamate (lGLTa), D-xylose (dXYLa), DL-lactate (LATa), acetate (ACEa), sodium citrate (CITa), glucoronate (GRTa), 2-keto-D-gluconate (2KGa), N-acetyl-glucosamine (NAGa), D-gluconate (dGNTa), L-proline (lPROa), Nitrate (NO3a), L-lysine-arylamidase (LysA), leucine-arylamidase (LeuA), arginine (ARG), tyrosine arylamidase (TyrA), gamma-glutamyl-transeferase (GGT), PNP-N-acetyl-BD-galactosaminidase1 (NAGA1), urease (URE), alpha-glucoside (AGLU), esculin hydrolysis (ESC)

### PCR amplification, sequencing, and phylogenetic analysis

All obtained data was compared with the GenBank databases using BLAST software (http://www.ncbi.nlm.nih.gov/blast). Table 6 presents the obtained sequence size, the GenBank accession number of the obtained and compared sequences, the identity yeast strain, the sequence identity, and the identified species. A phylogenetic tree and analysis have been performed to illustrate the genetic similarities and relativeness between the sequenced three *R. mucilaginosa* isolates obtained during the current study and another 27 major homologous selected from the GenBank database as shown in Supplementary Fig. 1.

**Table 6:** Summarization and tabulation of the sequencing obtained data

	Cluster 1	Cluster 2	Cluster 3
**The obtained sequence size**	609 bp	601 bp	607 bp
**The GenBank accession number of the obtained sequence**	PQ435489-PQ435490	PQ421573-PQ421574	PQ421577-PQ421578
**The GenBank accession number of the compared sequence**	KY218730.1	KM246181.1	JQ425392.1
**The identity yeast strain**	IIFCSW-B2	AM25	AUMC 7778
**Sequence identity**	99%	99%	99%
**The identified species**	*R. mucilaginosa*	*R. mucilaginosa*	*R. mucilaginosa*

The obtained sequencing results identified the representing three isolates as *R. mucilaginosa*

## Discussion

The result obtained from the current work is urgent evidence of the incrimination of *R. mucilaginosa* in certain clinical conditions among pet animals. Also, it sheds light on four main persistent points that must be clearly discussed and further studied in an insightful prospective manner: (1) The clinical potential and the seriousness of the existence or coexistence of *R. mucilaginosa* in active infection cases, (2) The talk-sharing role of *R. mucilaginosa* as an emerging opportunistic fungal pathogen to the already conceptualized cross-kingdoms talk between the bacterial pathogens, fungal pathogens, co-establishment in a susceptible host reflecting the underlying statuses including immune condition, physiological or pathological stressors or predisposition, previous recurrent or chronic willing clinical conditions, and microbiome integrity and diversity situation, (3) The antifungal susceptibility pattern of *R. mucilaginosa* focusing on resistance and the possible exaggeration of this panel into more complicated clinical situations in the shadow of the polymicrobial and co-microbial existence, and (4) The possible public health and zoonotic risks of *R. mucilaginosa* shedding the light on pet animals and animal-human companionship role in fasten the zoonotic impact of this emerging pathogen.

Many reports raised in a wide range of developing and developed countries studied the potential of *R. mucilaginosa* as a cause of disease and its recent millennium opportunistic emergence. Individual or collective case reports are mostly in human cases and with less contribution by animal cases [10, 47–54]. To the best of our knowledge, although there are many already reported animal rhodotorulosis cases, the current work is considered the first study focusing on figuring out the actual clinical role of *R. mucilaginosa* as a disease-causing agent in pet animals with a clear investigatory glimpse on the characteristics of the obtained isolates and their antifungal susceptibility profile, nature of the caused disease, the multi-predisposing factors of these cases, and the infection and post-infection establishment related and correlated micro-environmental circumstances.

Poly-microbial infections are of paramount significance because of the potential severity of complicated clinical manifestation possibilities. They also act as hindering obstacles against mono-microbial-specific treatment success, often associated with raised antimicrobial resistance treatment. The poly-microbial advantage to co-microbial infection persistence is reflected in the currently investigated cases as described in Table 1 as bacterial isolation was first obtained in all cases. Antibiotic sensitivity tests were performed separately for each isolate. The clinician’s tentative prescriptions were based on these presumptive results. Unfortunately, there was no proper case response reflecting the poly-microbial role in the overall microbial survival and persistence against treatments in all cases [55, 56]. As well as observed improvements were noticed after finishing the mycological laboratory investigation and the fungal co-infectious agent was identified and tested for its antifungal sensitivities as in Table 4 in association with correction and re-prescription of a combined curative treatment protocol. Although only three isolates have been sequenced which may be considered as a limitation in the current work, the obtained sequencing results identified the representing three isolates as *R. mucilaginosa* agreed 100% with the phenotypic identification including the primary isolation and identification, biochemical and post-culturing characterization, and VITEK 2 Compact Identification System.

Due to the multidrug resistance patterns of the obtained isolates, it is expected that treatment will be difficult if they find their way to infect humans in a zoonotic-saprozoic infection cycle. Unfortunately, few reports of drug susceptibility testing of *R. mucilaginosa* are based on standard antifungal susceptibility testing. Therefore, more studies are required in this field to find the points of acquiring resistance. Canine skin and ear microbiomes were investigated using next-generation sequencing assays, they did not detect *R. mucilaginosa* or any other *Rhodotorula* spp. from healthy and clinically affected dogs [57] and that reflects on the current study investigational finding regarding the pre-infection outdoor exposure/allowance of all involved cases, as a rich source of ubiquitous fungi including *Rhodotorula* spp. The complete absence of a predominant ear and cutaneous mycobiota member in pet animals, *Malassezia pachydermatis*, is an obvious clue reflecting a microbiota-disturbing effect of *R. mucilaginosa* and a dysbiotic relationship between both [58]. Also, it is worth focusing on the age, gender, and animal species and their relativeness to *R. mucilaginosa* incidence through the obtained results. *R. muciloginosa* incidence was higher in females than males, 77% and 33% respectively, higher in dogs than cats, 81% and 19% respectively, and seniors higher than adults and adults higher than puppies/kittens, 0% in puppies/kittens, 14.3% in adults, and 85.7% in seniors.

It is well established that long-term or repeated exposure to broad-spectrum antibiotics, impairment of epithelial barriers affected by chemotherapy, surgery, central venous catheters, and treatment with immunosuppressive agents such as corticosteroids, all are considered predispositions for the emergence of opportunistic pathogens [59]. Numerous virulence factors and adaptability mechanisms are activated because of the interaction between microbial pathogens and the host [60, 61]. These findings support the current work correlative findings between the successful *R. mucilaginosa* co-infection establishment and underlying managemental (poor nutritional regimen), medicinal (prolonged antibiotic and/or corticosteroid administration), physiological (elderly and/or pregnancy), and/or pathological (recurrent and/or chronic diseases) immune-impairment conditions that existed in all cases that reported as positive rhodotorulosis cases.

Prospectively, it is worth focusing on the co-existence of *R. mucilaginosa* with co-infectious bacterial and fungal pathogens, highlighting the attribution of this coexistence and a clear alteration in the routine scenario of the host-pathogen interfaces should be confessed. A more realistic involvement of the coinfection and its effect on the host immune system response and host-associated microbiota micro-environmental behavior should be adopted and investigated [59–71]. Drug-resistant *R. mucilaginosa* occurrence in the nasal passages and ear canals of pet cats and dogs carries a concern that the risk of zoonotic cases may emerge in an exponential increase pattern between companion animals and their caregivers.

## Conclusion

The current study represents the first elucidation of the predicted evolution of the emerging opportunistic fungal pathogen, *R. mucilaginosa*, focusing on the cross-kingdom-talks conceptualization as well as hitting the warning bell to be ready for any uprising microbial-based danger that may face humanity in the upcoming few years. Also, it is worth mentioning that controlling human contact with animal reservoirs helps to safeguard vulnerable groups and is an essential part of any prospective prevention strategy. To more accurately describe the burden, distribution, mortality, and socioeconomic effects of any possible neglected zoonotic or sapronotic potentials and to provide an integrated platform of prevention and control techniques, greater awareness-raising initiatives are required. Additional research investigations on the pathogenesis, expected risks, molecular and epidemiological tracing and follow-up, diagnostic techniques, as well as efficient and early therapeutic protocols are currently of importance. The current findings leave us with a very urgent question; Does *R. mucilaginosa* pose a public health risk or is it an exaggerated concern? Therefore, and due to the scarce contribution of the scientific community in this area of inquiry, further studies should be performed to ascertain the etiologic significance of different *Rhodotorula* spp. in other and more clinical diseases of humans and animals that may be incriminated in, currently and prospectively.

## Electronic supplementary material

Below is the link to the electronic supplementary material.

Supplementary Material 1

## Data Availability

All obtained data are presented in the manuscript. The obtained sequencing data are deposited in GenBank and presented by freely accessible accession numbers; PQ435489-PQ435490, PQ421573-PQ421574, and PQ421577-PQ421578.
