# Supplementary material for: Emergence of Rhodotorula mucilaginosa among pet animals: a possible public health risk on the move
Source: BMC Microbiol. 2025 May 7;25:273. doi: 10.1186/s12866-025-03894-9 (PMC12056995; doi:10.1186/s12866-025-03894-9)
Supplement: Supplementary file 1 — Supplementary Material 1 [file 12866_2025_3894_MOESM1_ESM.docx]

**Supplementary Figure 1:** Phylogenetic tree illustrates the genetic similarities and relativeness between the sequenced three *R. mucilaginosa* isolates obtained during the current study and another 27 major homologous selected from the GenBank database.

The evolutionary history was inferred using the Neighbor-Joining method [1]. The optimal tree with the sum of branch length = 0.06626072 is shown. The evolutionary distances were computed using the Maximum Composite Likelihood method [2] and are in the units of the number of base substitutions per site. This analysis involved 30 nucleotide sequences. All ambiguous positions were removed for each sequence pair (pairwise deletion option). There were a total of 1220 positions in the final dataset. Evolutionary analyses were conducted in MEGA X [3]

1. Saitou N. and Nei M. (**1987**). The neighbor-joining method: A new method for reconstructing phylogenetic trees. *Molecular Biology and Evolution* **4**:406-425.

2. Tamura K., Nei M., and Kumar S. (**2004**). Prospects for inferring very large phylogenies by using the neighbor-joining method. *Proceedings of the National Academy of Sciences*(*USA*) **101**:11030-11035.

3. Kumar S., Stecher G., Li M., Knyaz C., and Tamura K. (**2018**). MEGA X: Molecular Evolutionary Genetics Analysis across computing platforms. *Molecular Biology and Evolution* **35**:1547-1549.
